# Adult primary retroperitoneal cavernous hemangioma: a case report

**DOI:** 10.1186/1477-7819-10-261

**Published:** 2012-12-08

**Authors:** Hang He, Zunguo Du, Sijie Hao, Lie Yao, Feng Yang, Yang Di, Ji Li, Yongjian Jiang, Chen Jin, Deliang Fu

**Affiliations:** 1 Pancreatic Disease Institute, Department of Pancreatic Surgery, Huashan Hospital, Fudan University, Shanghai 200040, China; 2 Department of Pathology, Huashan Hospital, Fudan University, Shanghai 200040, China

**Keywords:** Cavernous hemangioma, Retroperitoneal, Primary

## Abstract

Primary retroperitoneal cavernous hemangioma (PRCH) in an adult is extremely rare. We report on the diagnosis and treatment of a patient with PRCH with subtle clinical features and atypical findings on imaging scans. A 38-year-old man was admitted to hospital with a 5-day history of epigastralgia after alcohol drinking. Using various imaging methods, we found a giant cyst-like retroperitoneal mass compressing the surrounding organs. Surgical resection of the tumor was performed, and the mass was found to be a cavernous hemangioma measuring 90 × 80 × 60 mm, with a thick fibrotic wall and extensive intracystic hemorrhage. Physicians should be aware that PRCH may mimic a cystic neoplasm, and that a large tumor size probably indicates intracystic hemorrhage. Surgical resection is a curative approach for PRCH.

## Background

Vascular tumors are non-epithelial tumors, which are more common in childhood. Hemangiomas are vascular tumors that rarely involve the retroperitoneal organs. The majority of reported retroperitoneal hemangiomas in adults originated from the kidneys, but hemangiomas can also originate from the adrenal glands and pancreas [1-3]. Primary retroperitoneal cavernous hemangioma is a rare type of hemangioma, and is unique in that it is separated from the surrounding organs. In the only report of PRCH in the literature, Haas *et al*. reported that PRCH was a solitary tumor with subtle clinical features [4]. Generally, hemangiomas are found when patients present with abdominal pain, hematuria, and melena, or are incidentally diagnosed by histopathological examination after resections of suspected masses identified by ultrasonography (US), computed tomography (CT), angiography, or magnetic resonance imaging (MRI). This paper reports a case of PRCH with extensive intracystic hemorrhage and atypical imaging features in an adult patient.

## Case presentation

A 38-year-old man presented with a 5-day history of dull epigastralgia after alcohol drinking, without radiating to back. There were no signs of other disorders, including fever, chills, jaundice, nausea, vomiting, melena, or hematuria at this first visit. The patient’s medical history was unremarkable. On physical examination no abnormal signs were seen, except for mild tenderness of the epigastrium. Laboratory studies, including tests for serum amylase, creatinine, alanine and aspartate aminotransferases, bilirubin, and urea nitrogen gave normal results.

**Figure 1:**
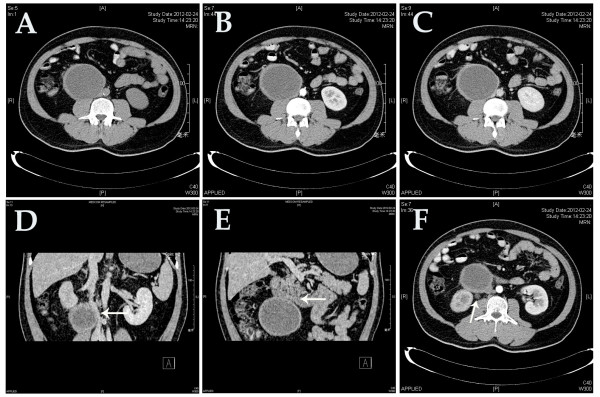
**Computed tomography scans.** (**A**–**C**) Plain scan, artery phase, and portal phase, respectively, showed a cyst-like and well-encapsulated tumor, with mild enhancement of the wall on portal phase. (**D**–**F**) Coronal and transverse sections showed that the tumor was compressing the inferior vena cava, duodenum, and right upper ureter respectively (white arrows).

Imaging studies were carried out. US showed a giant cystic mass in the right upper quadrant, accompanied by right hydronephrosis and right upper ureterectasia, but no additional abnormalities of the liver, cholecyst, pancreas, or urinary tract were found. Using enhanced CT, a cyst-like and well-circumscribed mass measuring 87 mm at the greatest diameter with low density (14.8 Hounsfield units) was found, which was adjacent to the inferior vena cava with its upper pole between the third part of the duodenum and the right renal hilum. Mild enhancement of the thick wall and consistent hypodensity within the inner component of the mass were seen during both the arterial and portal phases of CT. There was no evidence of feeding arteries from the surrounding organs or of adenopathy. The CT scan also confirmed the obstruction of the right upper urinary tract (Figure 1).

Further treatments including surgical intervention were suggested, but the patient didn’t want to receive any invasive treatments at this first visit. Medication such as antispasmodic was prescribed and scheduled follow-up was required. A month later, the patient was readmitted to our department as the symptoms continued. During this second visit, there were no other physical symptoms or signs, except for mild epigastralgia. Results of tests for serum tumor markers, including carcinoembryonic antigen, carbohydrate antigen 19-9 and α-fetoprotein, were all within normal ranges. Enhanced CT was repeated, again showing that the giant cystic mass was compressing the duodenum, inferior vena cava, and right urinary tract. The case was reviewed, and the mass was considered to be a benign isolated retroperitoneal lesion. However, the definite nature of the lesion could not be established preoperatively. Laparotomy was required because of the size of the lesion and also for relief of the pressure on the surrounding organs.

**Figure 2:**
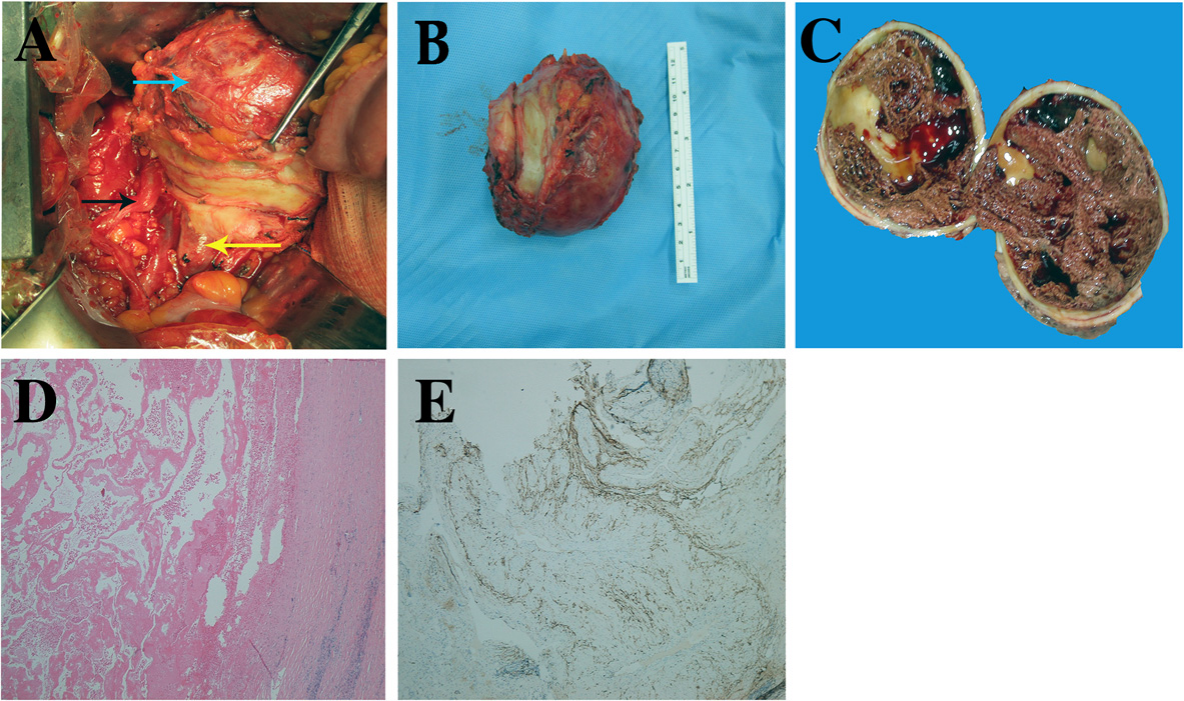
**Clinical and histological findings.** (**A**–**C**) Intra-operative, post-operative, and cross-slit findings of the tumor. (**A**) The blue, black and yellow arrows indicate tumor, right upper ureter, and inferior vena cava. respectively. (**D**,**E**) Stains confirmed the components of the tumor. (**D**) Hematoxylin and eosin, original magnification ×40; (E) CD34, original magnification × 40.

A vertical midline incision and extensive Kocher maneuver was used for the surgery. A giant tumor was found, lying inferior and posterior to the pancreatic process and the third part of the duodenum, inferior and anterior to the hilum of the right kidney, which was compressing anteriorly and laterally on the inferior vena cava, medially on the right upper ureter and posteriorly on the duodenum (Figure 2). No metastatic lesions were found in the peritoneum, abdominal organs, or pelvic organs. Radical resection of the tumor was completed in 3.5 hours, with an estimated blood loss of 200 ml. The feeding arteries of the tumor were found to originate from retroperitoneal tissue instead of from the abdominal artery or from other organs, and each vessel was ligated before the tumor was removed. There was no evidence intraoperatively of invasion of the inferior vena cava, ureter, renal capsule, pancreas, duodenum, or other surrounding organs. The post-operative period was uneventful, and the patient discharged home on day 9 after the operation. Resection of the tumor relieved the pressure on the neighboring structures and no obvious complications of the surrounding organs were found at the 3-month follow-up.

Examination of the mass showed it to be a cystic, well-encapsulated tumor, with a yellowish to tan color, measuring 90 × 80 × 60 mm (Figure 2). The tumor had a thick wall and spongy appearance, with large dilated spaces filled with clots and blood, and surrounded by multiple lacunas containing a light-yellow gelatinous liquid mixed with blood. Microscopically, the findings were consistent with a cavernous hemangioma (CH), with multiple vascular spaces of various sizes, lined by a single layer of flattened cells. Fibrosis and infiltration of inflammatory cells were seen in the tumor wall. Immunohistochemistry gave positive results for CD34 and vimentin staining, and negative results for cytokeratin.

## Discussion

Vascular lesions are composed of two major types of abnormalities: hemangioma and vascular malformation [5]. CH is a benign vascular tumor, commonly involving the skin or mucosa. Other visceral CHs originate from the liver, spleen, kidney, adrenal gland, and pancreas. In some childhood cases, the hemangiomas are accompanied by the Kasabach–Merritt phenomenon, and are considered to be a congenital abnormality [6]. Adult hemangiomas are distinct from pediatric hemangiomas, which proliferate during infancy and then involute slowly for several years, followed by eventual regression [7]. Adult CHs are uncommon in retroperitoneal organs, and adult PRCH is even rarer with only one case reported previously in the literatures [4].

Our patient was a middle-aged man. No gender predilection has been found for retroperitoneal CH [8,9], except for tumors in the adrenal gland [10]. The patient’s presenting symptoms were unremarkable, with the main symptom being dull abdominal pain, which would have been due to the slow-growing CH. However, large CHs can cause compression of surrounding organs and corresponding complications, such as the hydronephrosis and upper ureterectasia in our patient. Renal CHs are generally 10 to 20 mm in size, and patients usually report flank pain or asymptomatic hematuria [1,11], while patients with pancreatic CH usually report abdominal pain, back pain, or melena. By contrast, vague or asymptomatic presentations are common in patients with adrenal CH and PRCH [4,10]. Bleeding can be a serious complication of PRCH, ranging from asymptomatic anemia or thrombocytopenia to life-threatening bleeding, and is more subtle than the melena, hematemesis, or hematuria found with other types of CH [11,12]. Physical examination and laboratory studies usually provide no additional indications for PRCH.

We used several diagnostic imaging methods to characterize the tumor in this case. US showed a hypoechoic, homogeneous, and sharp-edged lesion, consistent with a cystic mass. On CT scans, a typical CH of the pancreas has strong contrast enhancement relative to the surrounding normal pancreas in the artery phase [9], and CH in the kidney is generally seen as a homogeneously enhanced mass relative to the renal cortex, with peripheral hypoenhancement in the portal phase of conventional CT imaging with intravenous contrast material [13]. Haas *et al*. reported enhancement of a soft-tissue mass by enhanced CT scan in their PRCH case [4]; however, in our case, CT showed an homogeneous, hypodense inner component of the tumor without enhancement during the artery or portal phase, except for mild enhancement on the wall of the mass. We consider that the atypical findings on the CT scan might be attributable to neovascularity, arteriovenous shunting, thrombosis, and hemorrhage, which slowed blood flow and therefore delayed the contrast material from filling the tumor [14]. In this case, the cyst-like tumor was considered preoperatively to be a benign lesion, separated from the surrounding organs, therefore angiography was not performed. Although the features of the tumor in this case were atypical on US or CT, we did not use MRI in the differential diagnosis; MRI might have been helpful to characterize the inner component of the tumor [4,8,14].

Our first impression in this case was of a cystic tumor originating from the retroperitoneum. For such cases, the differential diagnosis includes malignancies such as liposarcoma, malignant fibrous histiocytoma, leiomyosarcoma, and neuroblastoma, or benign lesions such as paraganglioma, neurofibroma, lipoma, teratoma, and neurilemoma [4]. Given that the tumor was localized and there was no evidence of invasion or metastasis to peripheral organs, we performed curative resection of the tumor to relieve the pressure on neighboring organs. No serious complications after surgery were seen in this case or in previously reported large retroperitoneal CHs [9,14]. Pathologically, the gross findings of the tumor included dilated spaces filled with blood, consistent with the typical appearance of CH. However, the thick wall seen in our patient’s tumor is rare in CHs, and this was distinct from lesions in the kidney, pancreas, adrenal gland, or even previously reported PRCH [4]. The size of CH in the kidney is usually around 10 to 20 cm [11], whereas our patient’s PRCH case was much large, as has been seen in some CHs arising from the adrenal glands (often > 100 mm) [10] or the pancreas (often > 50 mm) [9]. Microscopically, the inner components of the tumor consisted of variously sized vascular spaces lined by a single layer of flattened cells, which stained positive with CD34 and vimentin. CD31 and factor VII-related antigen are also informative markers for the diagnosis of CH [9,15]. In this case, there was inflammation and fibrotic thickening of the tumor wall, which probably prevented the contrast medium from filling the tumor on CT scan.

## Conclusion

In this study, we present a case of PRCH, a rare type of retroperitoneal CH, in an adult patient in our hospital. The tumor was separated from surrounding organs, thus it was diagnosed as a primary retroperitoneal CH [4]. The PRCH in this case had a thick fibrous wall and extensive intracystic hemorrhage, leading to the initial cyst-like appearance. The clinical features of PRCH may be more subtle than other types of CH and clinicians need to be alert to the possibility of PRCH as such tumors can grow to a very large size and cause serious complications. Surgical resection is a curative treatment for PRCH, which reduces the risk of hemorrhage and relieves the pressure on neighboring organs.

## Consent

Written informed consent was obtained from the patient for publication of this case report and any accompanying images. A copy of the written consent is available for review by the Editor-in-Chief of this journal.

## Competing interests

The authors declare that they have no competing interests.

## Authors' contributions

HH, HSJ, and DY collected the information, reviewed the literatures and wrote the manuscript. YL, YF, LJ, and JYJ participated in analyzed the data and interpreted the imaging features. DZG carried out the pathological studies. JC and FDL edited the article, and gave the final approval of the version to be published. All authors read and approved the final manuscript.
